# Rapid Integrated Healthcare Response for the First US Evacuees from Wuhan, China, During the COVID-19 Pandemic

**DOI:** 10.1017/dmp.2020.497

**Published:** 2020-12-22

**Authors:** Michael Mesisca, Geoff Leung, Jonelle Morris, Matthew Chang, Roderick Verbeck, Stephanie Loe, Jennifer Cruikshank, Arnold Tabuenca, Rhyan Miller, Cameron Kaiser, Kim Saruwatari, Andrew Pachon, Melanie M Randall

**Affiliations:** 1 Riverside University Health System Medical Center, Moreno Valley, CA, USA; 2 Riverside University Health System Behavioral Health, Riverside, CA, USA; 3 Riverside County Public Health, Riverside, CA, USA

**Keywords:** quarantine, disaster planning, emergency medicine, emergency services, psychiatric, epidemics

## Abstract

On January 29, 2020, a total of 195 US citizens were evacuated from the coronavirus disease 2019 (COVID-19) epidemic in Wuhan, China, to March Air Reserve Base in Riverside, California, and entered the first federally mandated quarantine in over 50 years. With less than 1-d notice, a multi-disciplinary team from Riverside County and Riverside University Health System in conjunction with local and federal agencies established on-site 24-h medical care and behavioral health support. This report details the coordinated efforts by multiple teams that took place to provide care for the passengers and to support the surrounding community.

On January 29, 2020, a total of 195 US citizens from Wuhan, China arrived at March Air Reserve Base (MARB) in Riverside County, California. All passengers had been potentially exposed to the novel coronavirus, severe acute respiratory syndrome coronavirus 2 (SARS-CoV-2), which was at epidemic levels in Wuhan. Our team at Riverside University Health System Medical Center (RUHS-MC), the local county hospital, had 11 h of advanced notice to establish medical operations and quarantine protocols. Over the next 14 d, from January 29 to February 11, the RUHS-MC provided full onsite medical and behavioral health services at MARB to all passengers during their mandated quarantine. Here, we outline our response and management of the first federally mandated quarantine in the United States since the 1960s.^1^

## Narrative

In January, the SARS-CoV-2 epidemic was increasing exponentially in China, and the US government began to evacuate its citizens from the Hubei province.^2^ At the time, there were no known cases in Riverside County and no local spread. There was also no ability to obtain SARS-CoV-2 testing locally. The flight had been scheduled to bring passengers to a neighboring county; however, due to considerations of security, privacy, and housing, the plane was redirected last-minute to Riverside County. MARB is an active reserve base located 4 miles from RUHS-MC, and while this was a civilian operation, the military agreed to house the evacuees in a dormitory on base at the request of the federal government. RUHS-MC is a public hospital serving 2.47 million residents of Riverside County and has a multi-disciplinary Disaster Medical Team (DMT) that responds to natural and human-caused disasters. The DMT has a long-standing collaboration with the county Department of Public Health (DPH) and Emergency Management Department on training, education, and disaster exercises, including a full-scale exercise annually in conjunction with MARB. The leaders of the DMT were accustomed to meeting and collaborating regularly and, thus, were well positioned to support federal and state agencies to deploy a full-scale medical response in the extremely short time frame.

There were multiple components to be considered in preparing for care of the evacuees. The quarantined passengers would have restricted movements but would require access to high-quality medical and mental health services. Given that SARS-CoV-2 had not yet spread locally, all efforts were made to provide care onsite at MARB to limit exposure to the public. The passengers consisted of a wide range of ages and comorbidities, including a newborn child, a pregnant woman, adolescents, and adults. Behavioral health needs that were considered included the impact of the epidemic in China, prolonged transport, enforced quarantine away from their homes, and the implications of returning to their local community postquarantine.

Before departure in Wuhan, the passengers were screened for fever and known symptoms of cough and dyspnea. They were screened again in Anchorage, Alaska, and before leaving Alaska for MARB. Upon arrival, the RUHS-MC and DPH teams assisted the Centers for Disease Control and Prevention (CDC) in testing of symptomatic individuals with basic laboratory testing, and oral-pharyngeal and nasal-pharyngeal SARS-CoV-2 testing. SARS-CoV-2 testing was performed on all 195 passengers within 48 h of arrival. DPH epidemiologists on-site supported the CDC in testing, patient tracking, and monitoring of symptoms.

To provide care for the next 14 d, the RUHS Department of Emergency Medicine, DMT, Mobile Health Clinic, Behavioral Health Mobile Crisis Response Team, and Riverside Emergency Management Department collaborated to mobilize resources to MARB. The RUHS-MC Mobile Health Clinic, an already established initiative, makes regular visits to the community, and is staffed by primary care advanced practice providers and physicians. This Mobile Health Clinic was expanded to include a board-certified emergency physician and emergency department registered nurse to be deployed to the base with 24/7 coverage. The team had all capabilities to address basic medical complaints, including electrocardiogram, cardiac monitoring, laceration care, splinting, and portable ultrasound. Because the base had no infirmary, RUHS-MC made its laboratory and pharmacy services fully available to the team by means of courier. Local law enforcement support by the Riverside Sheriff’s Department was instrumental in timely transport of medications, equipment, and personnel between the base and the medical center. Emergency medical services by American Medical Response transported 3 patients that were ultimately admitted to RUHS-MC for concerning symptoms. All security was provided by military base personnel.

A behavioral health support person was on-site from 8 am to 5 pm daily, consisting of a combination of psychiatrist, behavioral health service administrators, therapists, and substance use disorder counselors. A psychiatrist was available on call 24 h/d. The behavioral health focus was to provide mental health screening, alcohol and substance use screening, crisis therapy, social support, and daily therapeutic interactions with passengers. The team partnered with a local nongovernment organization to distribute donated toys, sporting equipment, clothing, slippers, hygiene products, and food that included a coffee and refreshment station. Items were placed in a dedicated tent named the “Free General Store” in which passengers were able to browse and shop free of charge. It was here that the behavioral health team performed much of their support, gaining the trust and confidence of passengers. Crisis debriefing groups were held in the last 2 d to address the anxiety surrounding return to their communities and concern of stigma or discrimination that has been previously described in China and during previous outbreaks.^3,4^

During the 2 wk, the mobile health team provided 149 medical care encounters consisting of 3 categories: routine primary care, moderate complex care, and high complex care (Table 1). The behavioral health team provided 84 formal mental health encounters, not including group therapy. During all encounters with evacuees, both medical and behavioral providers were in personal protective equipment consistent of N95 mask, surgical mask, face shield, cap, gown, and gloves. Three hospital transports were made to RUHS-MC, including a child with a fever and viral symptoms, a federal employee with reactive airway disease who developed bronchitis with mild hypoxemia, and an adult with cough and fever. All were directly admitted to negative pressure isolation rooms for observation, and all were eventually discharged after being stabilized and testing negative for SARS-CoV-2. After 14 d of quarantine, the passengers returned to their homes; none ultimately tested positive for SARS-CoV-2 during the quarantine period.

**Table 1.** MARB on-site medical clinic summary

Total patient medical encounters	149
Age	
Patients < 18 y old	13
Patients ≥ 65 y old	5
Medical complexity^a^	
Routine primary care	137
Moderate complexity care	7
High complexity care (not transported)	5
Hospital transports	3

Abbreviation: MARB, March Air Reserve Base.

a Care definitions: routine primary: vital signs, history and physical exam, reassessments, pregnancy testing; moderate complexity: first aid, wound care, laceration care, full pharmacy support; and high complexity: full lab testing, electrocardiogram, intravenous fluids.

## Discussion

The RUHS-MC team was able to provide on-the-spot medical and behavioral health care in the field for patients during a quickly evolving and changing epidemic. The team established collaboration with local and county governmental agencies to support a large-scale federal quarantine response on incredibly short notice. The ability to deliver high quality and volume of onsite care, with few medical transports off base, points to the significant and effective collaboration among the team. This response served to influence the model for repatriations of US citizens from Wuhan that followed.

Ultimately, the onsite advanced medical clinic served as an extension of the main regional medical center, allowing for straightforward staffing, testing, and treatment. Instead of having an outside agency operating the medical care and attempting to integrate with local hospital resources, the RUHS-MC team was able to circumvent these barriers with an intimate understanding of medical center workflows and established personal relationships. The providers were able to incorporate the clinic into the RUHS-MC electronic medical record, allowing for accurate documentation, laboratory and pharmacy ordering, and care coordination. The few patients that were transferred to the medical center were able to be prearrived and directly admitted, bypassing the emergency department.

There were more than 10 medical encounters per day, showing that people leaving an epidemic hot zone may require high use of health care. The average American accesses routine outpatient or emergency medical care once every 3.75 mo.^5,6^ Extrapolating the number of mobile health clinic visits, the average evacuee was accessing medical care once every 20 d. The higher use is likely multifactorial due to delayed care before travel and repatriation, limited access to health care in Wuhan Province due to the epidemic response, and exacerbations of chronic health conditions due to situational stressors.

The 24-h coverage by an onsite emergency physician proved to be a critically important intervention. There were several patient visits that would have resulted in transport off-base with potential exposure to the community and hospital. One evacuee had an intracranial ventriculoperitoneal shunt with dizziness and hypertension. Close serial exams by the physician were performed directly overnight, and the patient’s symptoms improved with acetaminophen and anxiety treatment. One evacuee had chest pain and was evaluated with serial electrocardiograms and 3 sets of cardiac enzymes. Feedback from passengers at the conclusion of quarantine was exceedingly positive, referencing the reassurance that their care was locally coordinated and always accessible. The most important outcome of expanded onsite care was that it minimized transports, exposure to the community, and mitigated the impact on local hospital resources.

While the use of the military base to house evacuees was a last-minute decision, it became a key component in the success of the project. Given the intense media interest at the time, MARB provided evacuees safety, security, and privacy. Housing included private restrooms, external entries and exits that limited indoor interactions and exposure, and also an outdoor play area for exercise. With yearly disaster drills conducted among MARB, the county DMT, and RUHS-MC, the leaders of these groups are well known to each other and familiar with working together on large-scale initiatives.

We hope that this experience can serve as a model to catalyze and promote the establishment of a multidisciplinary approach to disaster medical operations and pandemic quarantine planning. The rapid mobilization and deployment of onsite effective care highlights the importance of preparedness efforts that focus on integration between hospital partners, public health, behavioral health, local and federal government, law enforcement, and military facilities.
